# Site-specific Labeling of a Protein Lysine Residue By Novel Kinetic Labeling Combinatorial Libraries

**DOI:** 10.5936/csbj.201403001

**Published:** 2014-03-26

**Authors:** Allen Krantz, Arthur M Hanel, Ivona Strug, Andrzej Wilczynski, Jeremy J Wolff, Wolin Huang, Linda H Huang, Tina Settineri, Darren L Holmes, Margaret C Hardy, Dominique P Bridon

**Affiliations:** a Advanced Proteome Therapeutics Inc., 650 Albany Street, Suite 113, Boston, MA 02118, United States; b RedCell Inc., 270-B Littlefield Avenue, South San Francisco, CA, 94080, United States (Renamed ConjuChem LLC. Current address: 11755 Wilshire Blvd, Suite 2000, Los Angeles, CA 90025.); c Current address: EMD Millipore, 17 Cherry Hill Drive, Danvers, Massachusetts, 01923, United States; d Bruker Daltonics Inc., 40 Manning Road, Billerica, MA 01821, United States; e Current address: Thermo Fisher Scientific, 355 River Oaks Parkway, San Jose, CA 95134, United States; f Current address: Optivia Biotechnology Inc., 115 Constitution Drive, Suite 7, Menlo Park, CA 94025, United States

**Keywords:** Site-specific protein modification, Kinetic labeling libraries, Affinity labels, Lysine labeling, Combinatorial libraries, Peptide libraries

## Abstract

The first example of a kinetic labeling library designed to enable the discovery of affinity labels is presented. Each library component (1) consists of a variable peptidyl component linked to a biotinyl moiety by a 4-mercaptobenzoyl linker in thioester format. We demonstrate that an affinity label can be uncovered by measuring reaction rates between library pools and the protein target, human serum albumin (HSA) and identifying significant outliers. By choosing peptide functionality compatible with a potentially reactive thioester labeling entity, libraries can be screened in pools. It is noteworthy that a limited subset of amino acids (R, S, E, F, Y, l, M, W, and Q) that compose the affinity moiety is sufficient to produce rate variances that guide the discovery process. After two rounds of deconvolution, J-FLYEE-NH_2_ (7-E) emerges as a *bona fide* affinity label of HSA. Unlike known affinity labels, the affinity moiety is not retained in the protein product, but is extruded upon acylation of the protein. This feature affords a method of introducing various payloads, without extraneous elements, onto protein frameworks.

## Introduction

Bioconjugation involving the covalent linkage of small chemical entities to macromolecules has numerous applications [1]. Indeed, protein conjugation is central to the discovery and development of protein therapeutics. Targeted therapies, such as antibody-drug conjugates, are focal points, but present difficult challenges if high standards of purity and homogeneity are sought [2–6]. In essence, the latter depends upon the ability to attach a specific entity to the same spot on each protein molecule, i.e., in a site-specific manner [7]. Failure to achieve specific labeling of a native protein with one equivalent of a labeling entity, limits the prospect of obtaining a chemically homogeneous sample. For many applications, such as the development of protein therapeutics, it is important to avoid mixtures as each unique component of a mixture has its own individual therapeutic profile. Therefore, reliable methods of site-specific labeling are sorely needed to expand the limited repertoire of chemospecific protein modifications, so as to provide new materials that can be well-characterized, analytically.

For investigations of the therapeutic properties of drug conjugates of human serum albumin (HSA), we sought stable conjugates of high purity that were linked by amide bonds. HSA was to serve both as a scaffold and delivery vehicle for therapeutically relevant small molecule entities, because of its relatively long half-life, *in vivo* [8]. Yet, a major gap in chemical modification technologies of proteins, involves the lack of methods for quantitatively labeling lysines, site specifically [9]. Successful application of combinatorial libraries for the discovery of tight-binding ligands to protein targets, prompted us to devise methods for the discovery of chemospecific labeling agents by screening novel “bonding”, or “kinetic labeling libraries”. Versions of such libraries, that we report on herein, may be regarded as diverse collections of potential affinity labels that consist of amine-reactive peptidyl thioesters. Since the peptidyl functions were chiral and mutually distinct, our expectations were that labeling-rate variances would occur and relate primarily to the structure of the peptidyl function. We were guided, intuitively, by the notion that relatively fast rates (“kinetic affinity”) might be indicators of site-specificity, in analogy with high affinity/high specificity trends invoked by Eaton et al., for tight-binding ligands [10].

**Figure 1:**
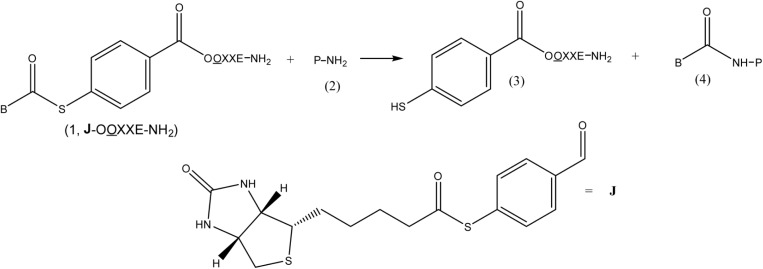
Reaction between a library member (1, **J**-OOXXE-NH_2_) and the target protein (2, P-NH_2_), involving transfer of the biotinyl function (B-C=O) with concomitant release of a 4-mercaptobenzoyl-pentapeptide (3).

The design motif for each library member is portrayed in Figure 1 and is comprised of three components: a variable peptidyl affinity group (OOXXE-NH_2_), a biotinyl moiety (B-C=O), and a 4-mercaptobenzoyl grouping. The latter component serves as a linker, as well as a constituent part of an amine-reactive thioester functionality. The split pool method of Furka was employed for pooling library members [11, 12]. Nine α-amino acids (arginine, serine, glutamate, glutamine, leucine, methionine, phenylalanine, tyrosine, tryptophan) were used to construct the variable peptidyl component, which was five residues in length. L-glutamate carboxamide was a constant feature at position 5, the C-terminal position. Two of the five positions were specifically defined in each pool by any one of nine α-amino acids. Nine amino acids of L-configuration were used to define the N-terminal position and were denoted by O; nine amino acids of D-configuration were used to define the contiguous 2-position and were denoted by O. The remaining two mixture positions were made up of the nine amino acids of L-configuration represented by X. The library was composed of 81 different mixtures, each of which contained 81 members, totaling 6561 peptidyl thioesters.

**Figure 2:**
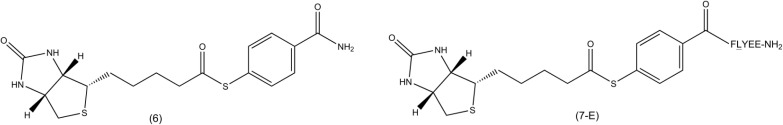
Structure of the fastest labeling entity of HSA (7-E, FLYEE-NH_2_) emerging from deconvolution of the pool of molecules of general structure, FLYOE-NH_2_. Note that leucine at position 2 of the peptide possesses the D-configuration. The standard of comparison, (6), is shown lacking a peptidyl group.

The overall reaction between a library member (1, **J**-OOXXE-NH_2_) and the target protein (2, P-NH_2_), as depicted in Figure 1, involves the transfer of the biotinyl (B-C=O) function to a protein amine, with concomitant release of a 4-mercaptobenzoyl-pentapeptide (3). Thus, in principle, both products from the reaction with HSA, afford a measure of deacylation rates of biotinylated substrates (1), but only the formation of biotinylated protein (4) is necessarily related to the labeling reactions. Accordingly, an ELISA assay (see Experimental) was designed to measure the extent of incorporation of the biotinyl moiety within the protein for each library pool. After testing each of the 81 mixtures in the library for their ability to label HSA with biotin, an iterative screening and synthesis process was carried out on the most active pool. By measuring the kinetics of sub-libraries, J-F-L-O-X-E-NH_2_ (5), and a standard, (6), lacking a peptidyl affinity group, a *bona fide* affinity label, (7-E), was ultimately revealed [13].

## Experimental Procedures

### Materials

Reagents used in ELISA determinations were from Sigma Chemical Co., St. Louis MO, except where otherwise noted. HSA for screening libraries was purchased from Calbiochem, La Jolla, CA. For enzymatic digestions, stoichiometric experiments, and related mass spectrometry experiments, albumins were from Sigma-Aldrich, as were dithiothreitol and iodoacetamide. Amicon filters were from EMD Millipore Corp., Billerica, MA. Enzymatic digestions were performed according to protocols suggested by the manufacturer (Princeton Separations, Adelphia, NJ). Library syntheses utilized Fmoc-protected amino acids and Rink Amide MBHA resin from NovaBiochem, San Diego, CA, but Fmoc-Tyr-(t-Bu)-OH was from Chem-Impex Int., Inc., Wood Dale, IL. Coupling agents were obtained from Aldrich Chemical Co, Milwaukee, WI. The syntheses of J-FLYEE-NH_2_ (7-E) and J-FLYEE-NH_2_ (8) for stoichiometric experiments utilized reagents from AnaSpec Inc., Fremont, CA. 4-mercaptobenzoic acid (85%) was obtained from Toronto Research Chemicals, Toronto, ON.

*Preparation of 4-(9’-Fluorenylmethylthio)benzoic acid:* To a 0° C. suspension of 3.40 g 4-mercaptobenzoic acid (18.7 mmol) in 60 mL N, N-dimethylformamide (DMF), was added 4.00 g 9-fluorenylmethyl chloride [14]. The reaction was allowed to slowly warm to RT and then stirred for 16 h. The cloudy yellow-brown solution was washed with hexanes (3x50 mL), diluted with 50 mL 1M HCl and extracted with ethyl acetate (3x50 mL), washed with 50 mL sat. aq. NaCl, dried with anhyd. MgSO_4_, filtered, and concentrated via rotary evaporation to afford a yellow-brown oil. This oil was then dissolved in 20 mL MeOH, cooled to -20° C. and the resulting crystals were collected by filtration, washed with ice cold MeOH, and dried to afford 5.59 g of *4-(9’-fluorenylmethylthio)benzoic acid* as a white solid (13.0 mmol, 70%).

### Synthesis of Kinetic Labeling Libraries

*General Considerations:* Eighty-one library pools, each containing 81 distinct pentapeptides, were synthesized consisting of a glutamic acid carboxamide at the C-terminal with the N-terminal connected to a biotinyl moiety via a 4-mercaptobenzoyl linker in thioester format. The N-terminal and the contiguous position in each peptide mixture were individually and specifically defined by nine amino acids (Arg, Glu, Gln, leu, Met, Phe, Ser, Tyr, Trp). Only amino acids of D-configuration were employed at the 2-position of the peptide. All other amino acids were of the L-configuration. Defined positions occupied by L- and D-amino acids are represented by O and O, respectively; mixture positions are represented by X. The eighty-one pools (OO= 9x9) are represented by the formulae: OOXXE-NH_2_. The eighty-one separate mixtures making up each library pool each contain 81 (9x9) sequences. The eighty-one library pools, when used in concert, constitute a kinetic labeling library consisting of 6561 biotinylated peptidyl thioesters (OOXX=9x9x9x9).

*Preparation of 81 pools of 81 peptides:* Multiple peptide syntheses were performed on a Rink amide 4-methylbenzhydrylamine (MBHA) resin with a Gilson AMS 422 Multiple Peptide Synthesizer using standard Merrifield SPPS methods and Fmoc/HBTU/HOBt chemistry. Coupling reactions were repeated until a negative ninhydrin test was obtained, after which the resin was washed repeatedly with DMF, followed by removal of the Fmoc group by the addition of 20% piperidine in N-methylpyrrolidinone (NMP) twice, 2 minutes and 15 minutes, respectively, at room temperature, followed by several NMP washes. Preparative HPLC was performed on a 21.4.x 250 mm C_18_ reverse phase column using 5-60% B (0.045% TFA in H_2_O and 0.045% TFA in CH_3_CN) gradient elution. LC/MS results were obtained using electrospray ionization on a Perkin Elmer Sciex API300 Mass Spectrometer with a 1.0 x.250 mm C_18_ reverse phase protein/peptide column using 0-70% B (0.045% TFA in H_2_O and 0.045% TFA in CH_3_CN) gradient elution.

Initially, Fmoc-Glu(OtBu)-OH was coupled to an identical amount of resin support in each of 9 reaction vessels by treating a DMF solution of the amino acid (4 equivalents), with o-benzotriazol-1-yl-N,N,N’,N’-tetramethyl-uronium hexafluoro-phosphate (HBTU) (4 equivalents), 1-hydroxybenzotriazole (HOBt) (4 equivalents) and diisopropylethylamine (DIEA) (8 equivalents), for 2 hours at room temperature (coupling conditions that were used throughout, unless otherwise noted). After removal of the Fmoc group, each of the nine different, Fmoc-protected, L-amino acids were then individually linked to an identical amount of resin in all 9 reaction vessels by coupling reactions that were carried out as above. The resins from each of the nine independent and distinct pools of dipeptides generated by this coupling were mixed and then divided into identical pools giving rise to nine identical pools (Fmoc-X-Glu-NH-Resin), each consisting of nine distinct dipeptides linked to the resin support. After removal of the Fmoc protecting groups, the resins were coupled to the nine different Fmoc-protected L-amino acids as above, were then mixed, and divided into identical pools, producing nine identical pools, each consisting of 81 distinct tripeptides (Fmoc-X-X-Glu-NH-Resin), linked to the resin support. Following removal of the Fmoc-protecting groups, nine different amino acids of D-configuration were coupled to the resins as described above. This coupling generated nine equimolar, independent and distinct pools of tetrapeptides linked to the resin support. Each of the resulting pools contains 81 distinct tetrapeptides that differ only with respect to the amino acid at position 4 (Fmoc-O-X-X-Glu-NH-Resin), producing a total of (81 x 9)=729 distinct tetrapeptides.

Each of the nine distinct pools of tetrapeptides was then divided into a set of nine identical pools without mixing the pools before dividing, giving rise to nine mutually distinct sets, each containing nine identical pools (81 pools total), with each pool containing 81 tetrapeptides. The step of splitting the pools without mixing permits the fourth and fifth amino acids coupled to the resin to be defined. After removing the Fmoc-protecting group, each of the nine identical pools within a set was then coupled with a mutually exclusive Fmoc-protected L-amino acid. This fifth round of coupling generates eighty-one equimolar, independent, and distinct pools of pentapeptides (Fmoc-O-O-X-X-Glu-NH-resin) linked to a resin support, totaling 81 pools x 81 pentapeptides = 6561 distinct entities.

After removal of the Fmoc-protecting group, each of the eighty-one pools was coupled to 4-(9’-fluorenylmethylthio)benzoic acid, followed by removal of the fluorenylmethyl protecting group. Each of the eighty-one pools capped with 4-mercaptobenzoyl was then coupled to biotin by adding to the resin a DMF solution of biotin (5 equivalents), HBTU (5 equivalents), HOBt (5 equivalents) and DIEA (10 equivalents). The reactions were allowed to proceed for 2 hours at room temperature and then repeated to ensure completion.

Cleavage and purification of the kinetic labeling libraries were performed as follows. The peptidyl resin mixtures in each of the 81 pools were dried and then independently treated with trifluoroacetic acid (TFA)/H_2_O (95/5, v/v) for 1.5 hours. The peptide/TFA solutions were then precipitated and washed with ether, after which the precipitates were suspended in 0.045% TFA in water and lyophilized, to yield the completely constructed and dried kinetic labeling library, which is substantially stable under normal storage conditions as determined by mass spectral analysis.

### Screening of Human Serum Albumin (HSA) against the Kinetic Labeling Library (81 pools)

*Capture Plate Preparation:* The evening before transfer of the quenched library (*vide infra*) for capture of the biotinylated fraction, twelve 96-well plates (NUNC's F96 Certified Maxisorp, Cole-Palmer, Vernon Hills, IL) were coated with rabbit anti-human serum albumin IgG (Boehringer Mannheim Biochemicals’ 605 001) by diluting the antibody 5000-fold into phosphate buffered saline (PBS, 10 mM Pi, pH 7.4, 138 mM NaC1, 2.7 mM KC1), and dispensing 0.1 mL of this solution into each well of each plate. The plates were covered, stacked and placed at 4 °C overnight. The following morning, the plates were emptied, and 0.2 mL of a solution of 1% (w/v) bovine serum albumin was added to the wells of each plate and left at room temperature (∼22-23°C) until just before use (∼4 h). Prior to transfer of the library pools, the plates were emptied and washed five times with PBS to prepare the plates for capture.

*Reactions of HSA with 81 Pools:* For triplicate measurements of hydroxylamine-quenched reactions and background mixtures of library pools in PBS, each of the 81 pools of the kinetic labeling library were dispensed into six wells of polypropylene plates (NUNC's heat resistant plate, Cole-Palmer, Vernon Hills, IL) at a concentration of 4.0 mM (based on total library member concentration) in DMSO in a total dispensed volume of 0.002 mL and then stored at -80 °C until used. A solution of 10.2 microg/mL HSA (Calbiochem, La Jolla, CA) in PBS was used to initiate the labeling reactions by adding 0.098 mL to each well to provide 0.08 mM/pool and 10 microg/mL (150 nM) HSA. After 1 min., 0.01 mL of 0.5 M hydroxylamine in water, pH 7.4, at room temperature (∼22 °C), was added with mixing to quench the reactions (45 sec). Each of the triplicate reactions was staggered at 15 sec intervals to facilitate uniform processing. Background reactions were initiated by the addition of 0.098 mL of a solution of 0.051 mM hydroxylamine, pH 7.4 to each well, and after one minute, 0.01 mL of a solution of 0.1 mg/mL HSA was added. In this manner each well of the plate had the same final composition with respect to the concentrations of HSA and hydroxylamine. Note that the time of quenching in the presence of hydroxylamine alone is 1 min., and after time t=1 min. both HSA and hydroxylamine are present.

*Binding of HSA to Capture Plates:* The extent of biotinylation of HSA was measured using the capture antibody method and the prepared plates that are described above. The quenched reaction mixtures and background mixtures in portions of 0.10 mL were then transferred directly to the washed wells of the capture plates which contained 0.01 mL of a solution of PBS consisting of 0.5% (v/v) Tween 20 and 1% BSA (w/v), to give 0.091% BSA and 0.046% Tween 20, a 10 to 11% dilution of the mixtures. The top plates of the two stacks were covered while the other plates of the stack were covered by the plate above, and the stacks were left at room temperature for 2 h to permit the binding of labeled HSA to the immobilized antibody. The plates were then emptied and washed ten times by the dunk and chuck method of washing that is described above.

*Detection of Biotinylated HSA:* Biotin was detected by the avidin-horseradish peroxidase enzymatic method as follows. To the wells of each plate was added 0.1 mL of a solution of 120 mL of General Conjugate Diluent (Medix Biotech Inc., San Carlos, CA) containing 0.24 mL of peroxide conjugate strepavidin (Jackson ImmunoResearch Laboratories, Inc. (West Grove, PA). This solution was incubated for 30 min. at room temperature, and the plates were then emptied and washed ten times by the dunk and chuck method. A solution (125 mL) of substrates for the peroxidase was prepared by dissolving o-phenylenediamine (OPD, 0.605 g) at a concentration of 5 mg/mL and adding 0.065 mL of 30% hydrogen peroxide in 125 mL of 0.111 M sodium phosphate dibasic, 0.044M citric acid, pH 5.4, to give 0.015% hydrogen peroxide. Substrates were added to the wells of each plate by dispensing 0.1 mL of the solution per well. The color was allowed to develop for 15 min., and the peroxidase reactions were then terminated by the addition of 0.1 mL of 2 N sulfuric acid (VWR). The plates were immediately read at 490 nm using a Molecular Devices SpectraMax 250 plate reader. The values of the absorbances of the individual wells were exported directly into a Microsoft EXCEL spreadsheet. The reactions of HSA with pools measured as ΔOD values were converted to sort values according to the equation: Average Reaction Value-(2 x s.d.)/Average Quench Value.

*Kinetics and Deconvolution Using S-Biotin-4-mercaptobenzoyl-F-L-O-X-E-NH*_*2*_ (5) *Sub-libraries:* Nine pools, each containing nine distinct pentapeptides, were synthesized using SPPS protocols analogous to those above. Each pool consisted of a glutamic acid carboxamide at the C-terminal with the N-terminal connected to a biotinyl moiety via a 4-mercaptobenzoyl linker. The N-terminal and the contiguous position in each peptide mixture were specifically defined by L-phenylalanine at the N-terminus and D-leucine at the 2-position. The 3-position was specifically and uniquely defined, in each pool by one of the nine amino acids of L-configuration. The mixture position-4 of the peptidyl thioester was randomly occupied by nine amino acids of L-configuration. Reactions of each pool were conducted in the PBS media above, with the total peptidyl thioester concentration at 0.080 mM (0.009 mM per peptide) in the presence of 150 nM HSA in a total volume of 1 mL. Labeling reactions were initiated by the addition of library members in DMSO, and 0.1 mL aliquots were withdrawn at time t = 1, 2, 3, 4, 5, 6, 7, 8 and 9 min. and mixed with 0.01 mL of 0.5 M hydroxylamine. The plates were shaken for 10 seconds after the addition of reaction mixture to quench. The reaction of the S-biotinyl-4-mercaptobenzamide (6) was monitored under identical conditions. Binding of HSA to capture plates and the detection of the conjugated biotin moiety by the strepavidin conjugate were performed essentially as above. Background reactions were also conducted as above. HSA was added either 5 sec or 15 min. after quenching with no significant differences in the resulting ΔOD values.

*Kinetics of Biotinylation of HSA with Constituent Peptidyl Thioesters of the J-FLYXE-NH*_*2*_ *Pool (7):* The nine individual peptidyl thioesters, with X designating the L-amino acids used above, were synthesized using the standard SPPS protocol. (However, biotinylation employed biotin (2eq), PyBOP (2eq) and diisopropylethylamine (4eq), 3x20 min at room temperature.) Reactions of each peptidyl thioester at a concentration of 0.080 mM were conducted in PBS media above, in the presence of 150 nM HSA. Background reactions and reaction of the S-biotinyl-4-mercaptobenzamide (6), the binding of HSA to capture plates, and the detection of biotin by the strepavidin conjugate, were performed essentially as above.

*Mass spectrometry studies:* Intact HSA and pig serum albumin, treated with J-FLYEE-NH_2_ (7-E), were analyzed on a solariX FTMS (Bruker Daltonics) equipped with a 12T actively shielded refrigerated magnet. The analyzed solutions were infused directly via ESI at a flow rate of 2 µL per min. Stoichiometric experiments of HSA or pig serum albumin (PSA) were carried out with HSA or PSA and (7-E) at 0.2 mM. Enzyme digests were divided for FTMS and MALDI analysis. Digested material was separated on a reverse phase column (Dionex C18 75 um x 15 cm) and delivered to FTMS (solariX, Bruker Daltonics) at 300 nL/min****. MALDI analysis was performed on an Ultraflex instrument (Bruker Daltonics). To enhance the MALDI analysis, digested material was divided into 12 fractions, on a BioBasic C18 RP column (100 x 2.1 mm) from Thermo Scientific) utilizing a gradient from 2 to 90% AcCN for 60 minutes. Each fraction was lyophilized and then solubilized in 2 µL of 75% AcCN containing 0.1% TFA, mixed with a-cyano-4-hydroxycinnamic acid (Bruker Daltonics), and spotted on MALDI target plates. MS data for peptides produced by each enzyme was collected in reflector mode. Peptide mass fingerprinting was performed using BioTools™; precursor ions identified as possible HSA fragments, specific to a given enzyme, were subjected to MSMS analysis.

## Results

**Figure 3:**
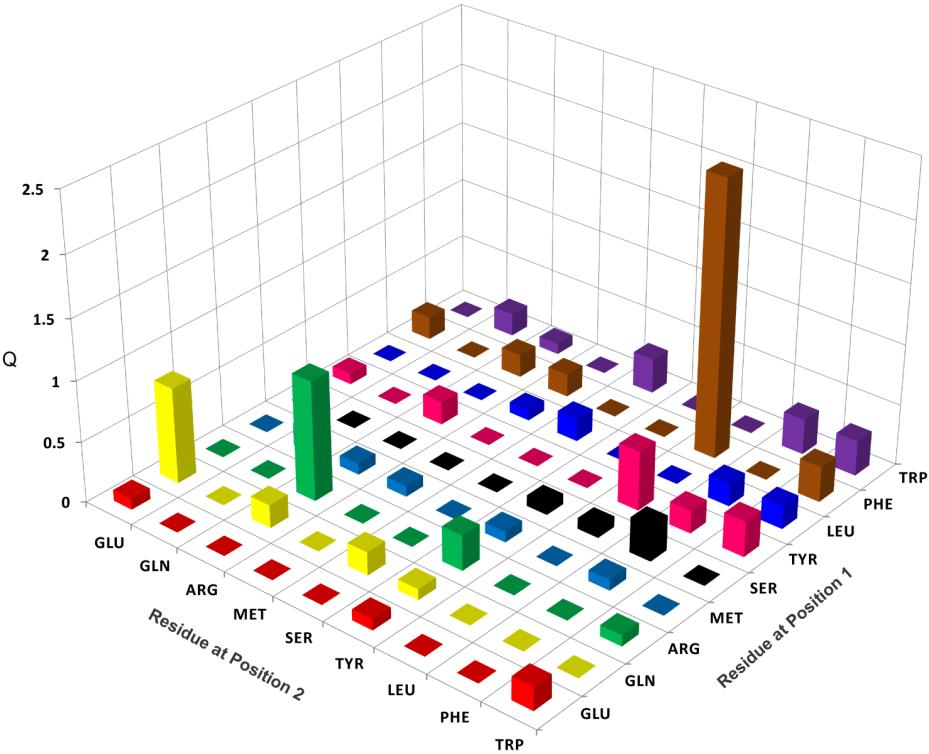
Results of treating human serum albumin for one minute with each of 81 pools, of general structure J-OOXXE-NH_2_, followed by quenching with 50 mM hydroxylamine. Sort values derived from ELISA ΔOD determinations (see experimental) are plotted on the z-axis for each pool, after subtracting the average sort value, 1.21. Thus Q (Y-axis) represents a pools’ sort value minus the average sort value of a library member. Residues at positions 1 and 2, are of the L- and D-configurations, respectively.

The results of treating human serum albumin with each of 81 pools of general structure (1) J-OOXX-NH_2_, for one minute, followed by quenching with 50 mM hydroxylamine, and then evaluating biotin labeling using an ELISA format, are shown in Figure 3. Sort values (see experimental) were calculated and plotted for each pool in the histogram in Figure 3, after subtracting the average sort value, 1.21. Thus, all pools above baseline in the Figure 3 incorporate a biotin moiety covalently, to a greater extent than pools with average or lower labeling rates. The pool of 81 defined by L-Phe at the 1-position, and D-Leu at the 2-position (J-FLXXE-NH_2_ (8)) was the major outlier with a sort value of 3.5.

The FLXXE-NH_2_ pool (8) was resynthesized as a sub-library containing nine mutually distinct pools of nine mutually distinct peptides, wherein each such pool possessed the general structure, J-FLOXE-NH_2_ (5). Thus, along with the F, L and E amino acids, position three (O_3_) was now specifically defined and was a distinguishing feature of each pool. The remaining position 2 was made up of mixtures of the nine amino acids (X).

**Figure 4:**
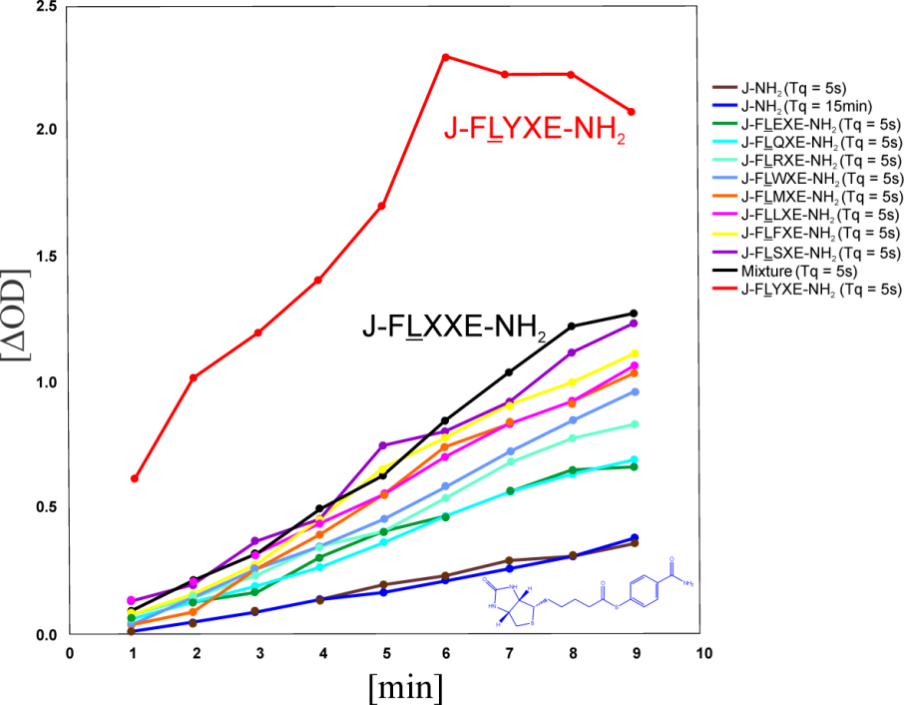
Results of a time course in which HSA was treated with each of nine pools of general structure J-FLOXE-NH_2_ (5). The kinetic data are compared against a standard lacking a peptidyl affinity group (6), and the master pool of 81, denoted by J-FLXXE-NH2 (black plot). Only the J-FLYXE-NH_2_ pool, (5-Y, red plot) substantially exceeded the rate of labeling of the master pool. (T_q_ refers to the time HSA was added to the quenched background reaction).

Figure 4 shows the results of a time course in which HSA was treated with each of the nine pools corresponding to (5). J-FLYXE-NH_2_ (5-Y), emerged as the fastest pool to label HSA. The nine individual members of J-FLYXE-NH_2_ were then synthesized and incubated separately with HSA. Although the rate differences were not dramatic, J-FLYEE-NH_2_ (7-E), proved to be the molecule which transferred biotin to HSA most rapidly (Table 1).

**Table 1:** Labeling Rates of HSA with Entities Comprising J-FLYXE-NH_2_ (7).

Biotin Substrates	Relative Rate
B-(C=O)-S-φ-p-CONH_2_ (6)	1 (0.081 OD/min)
Mixture of 9 (J-FLYXE-NH_2_)	10
J-FLYEE-NH_2_ (7-E)	13
J-FLYME-NH_2_ (7-M)	6
J-FLYSE-NH_2_ (7-S)	6
J-FLYQE-NH_2_ (7-Q)	5
J-FLYRE-NH_2_ (7-R)	5
J-FLYWE-NH_2_ (7-W)	7
J-FLYLE-NH_2_ (7-L)	6
J-FLYYE-NH_2_ (7-Y)	4
J-FLYFE-NH_2_ (7-F)	3

**Figure 5:**
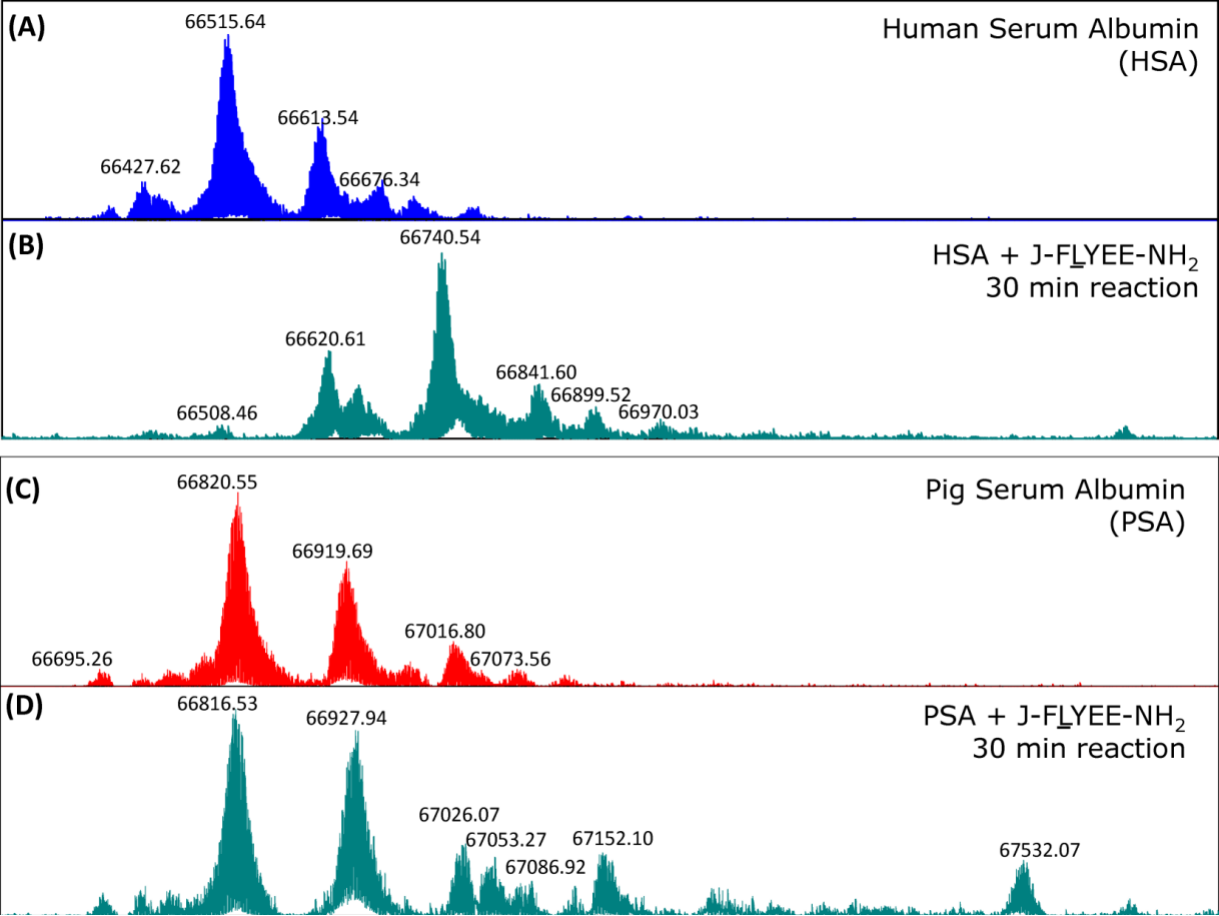
High resolution FTMS spectra of (A) native HSA, (B) HSA treated with one equivalent of J-FLYEE-NH_2_ (7-E), (C), porcine serum albumin (PSA), (D) PSA treated with one equivalent of (7-E). These stoichiometric experiments of HSA and pig serum albumin (PSA) were carried out with HSA or PSA and (7-E) at 0.2 mM.

To elucidate the labeling pattern under stoichiometric conditions, HSA was treated with one equivalent of J-FLYEE-NH_2_ (7-E). Incubation resulted in uptake of one equivalent of the biotinyl moiety, yielding monolabeled protein. The current study utilized native HSA containing varying degrees of post-translational modifications and genetic variants with molecular weight components ranging from 66,420 to 66,800 Da (Figure 5A). As observed with high resolution FTMS, treatment of native HSA with (7-E) for 30 minutes, resulted in a shift of the entire HSA mass population by essentially 228 Da, corresponding to the attachment of a single biotinyl moiety (Figure 5B).

A determination of the site of attack of native HSA, upon treatment with one equivalent of (7-E), was then pursued. Mono-labeled HSA was digested using each of the following enzymes: trypsin, chymotrypsin, Lys C, and Glu C. Digested material was analyzed by LC-FTMS and MALDI, which were used after off-line RP HPLC fractionation. The combined results provided coverage for 51 of the 60 amines present in HSA (see Table 2). Analysis of the data from the enzyme digests (Table 2) showed that trypsin delivered structural information on 30 lysine residues and the N-terminal amine, chymotrypsin allowed positive identification of 27 label-free lysine residues, and Lys C yielded structural information bearing on 33 amines, including fragment (182-195) in which K190 had been biotinylated. Interestingly, only one of the residues found in fragments produced by Glu C was detected in digests produced by trypsin and chymotrypsin. Twenty amine residues identified in the Lys C digest were also found in the tryptic digest, whereas only fifteen overlapped with the data from the chymotrypsin digest. There were nine lysines missing in the combined data from all four digests (K137, K162, K199, K205, K212, K436, K439, K524 and K560). K137, K162 and K199 would be detectable in digests produced by trypsin, chymotrypsin, and Lys C, only if they were modified, or in the event of an enzyme-missed cleavage site.

**Table 2:** N-terminal and lysine residues detected by mass spectrometry analysis of digests of HSA labeled by one equivalent of J-FLYEE-NH_2_ (7-E).‡

position	digest	position	digest	position	digest	position	digest
**N-teminus**	1	**K181**	1	**K317**	2	**K475**	3
**K4**	1, 2	**K190**	1, 3	**K323**	2, 3	**K500**	1, 2, 3
**K12**	2, 3	**K195**	3	**K351**	3	**K519**	3
**K20**	1, 3	**K199**	missing	**K359**	1, 2, 3	**K524**	missing
**K41**	2, 3	**K205**	missing	**K372**	1, 3	**K525**	1
**K51**	1, 3	**K212**	missing	**K378**	1, 2	**K534**	1, 2, 3
**K64**	1, 3	**K225**	2	**K389**	1, 2, 3	**K536**	2
**K73**	1, 3	**K233**	1, 3	**K402**	1, 3	**K538**	2
**K93**	1, 3	**K240**	1	**K413**	2, 3	**K541**	2
**K106**	1, 2, 3	**K262**	1, 2	**K414**	1, 2, 3	**K545**	2
**K136**	3	**K274**	1, 2	**K432**	3	**K557**	3
**K137**	missing	**K276**	1, 2	**K436**	missing	**K560**	missing
**K159**	3	**K281**	1, 2	**K439**	missing	**K564**	1
**K162**	missing	**K286**	1, 2, 4	**K444**	4	**K573**	1, 2, 3
**K174**	1, 2, 3	**K313**	1, 2, 3	**K466**	3	**K574**	1, 2

‡ 1 - trypsin; 2 - chymotrypsin; 3 - Lys C; 4 - Glu C

**Figure 6:**
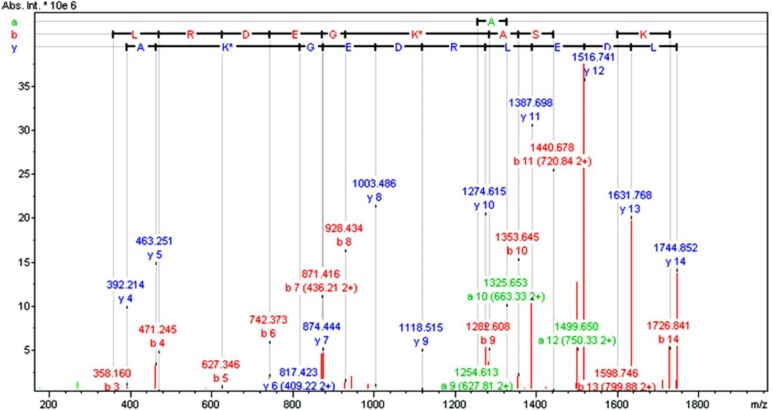
Evidence for biotin attachment at Lys-190, after HSA treatment with on equivalent of J-FLYEE-NH_2_ (7-E). CID product ion spectrum from the precursor ion of m/z 872.931 (+2) present in LC-FTMS analysis of labeled HSA that has been digested with Lys C.

Data obtained for the HSA fragment 182-195 (LDELRDEGK*ASSAK; MW 1744.853) provide compelling evidence that the protein has been biotinylated selectively at position K190. (K* represents biotinylated lysine 190 in fragment above and in Figure 6). Sound overlap of b and y ions in the fragmentation pattern of precursor ion m/z 872.931 (+2), supports the finding that the only labeled residue produced by treatment of native HSA with (7-E) is lysine at position 190 (Figure 6).

That (7-E) site-specifically labels HSA at Lys 190, is further supported by the results of treating serum albumins from non-human species, in which a residue, other than lysine, occupies position 190 [15]. For example, pig serum albumin, which contains leucine, a non-nucleophilic residue, at position 190, exhibits little tendency, under stoichiometric conditions, to react with (7-E) after 1 hr, as judged by mass spectrometry (Figures 5C and D). The mass spectrum shows no evidence of specific labeling of HSA at longer reaction times, reflecting the absence of both Lys 190, and a comparable environment responsible for the accelerated rate of reaction of (7-E) with HSA.

**Figure 7:**
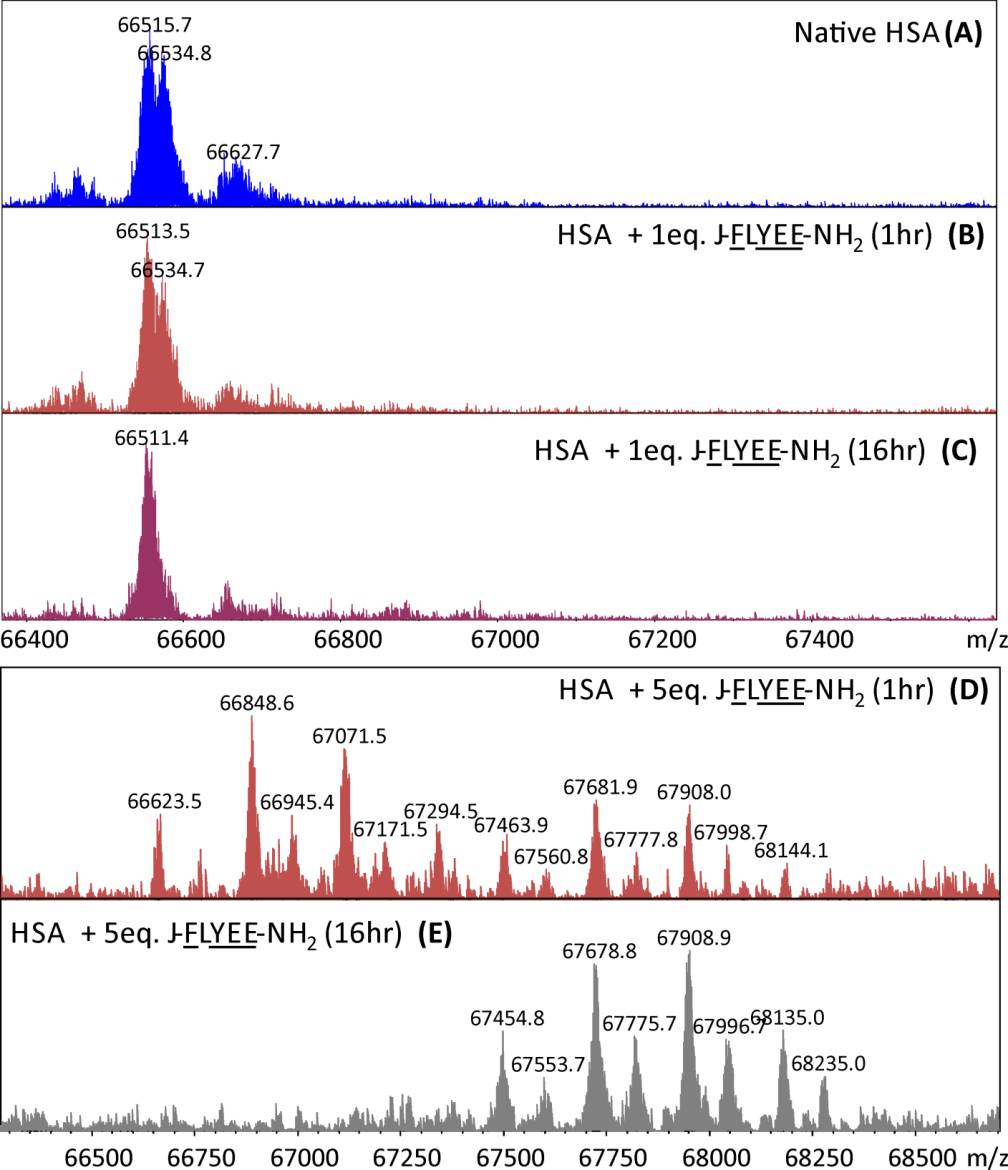
(A) High resolution FTMS spectra of native HSA, (B) HSA treated with one equivalent of J-FLYEE-NH_2_ (9) after 1 hr, and (C) after 16 hr, (D) HSA treated with five equivalents of (9) after 1 hr, and (E) after 16 hr.

Further evidence that (7-E) is likely complementary to the labeled site containing Lys-190, is obtained by incubating HSA with an equivalent of J-FLYEE-NH_2_ (9), containing a component peptide, enantiomeric to (7-E). No discernible reaction can be observed after 1 hour, or overnight (Figure 7). Monitoring higher concentrations of the enantiomer reveals complex spectra lacking a detectable dominant product.

## Discussion

### Rationale for the Concept of Kinetic Labeling Libraries

**Figure 8:**
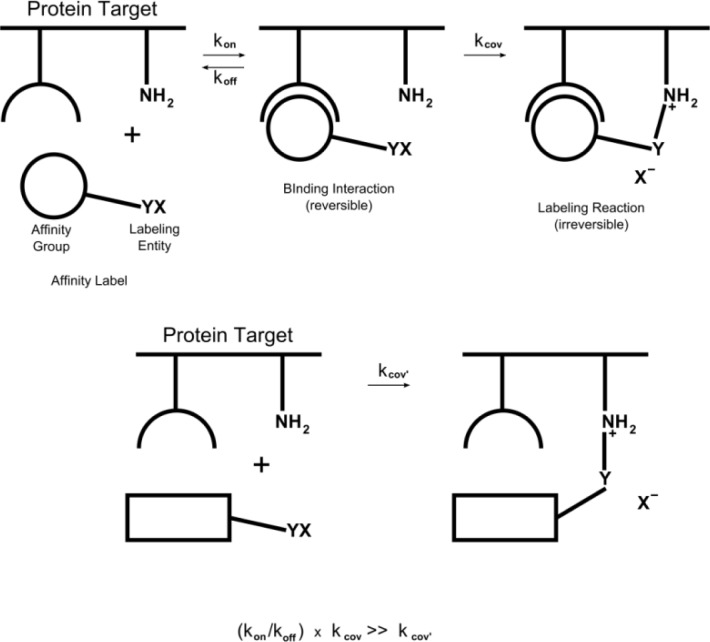
Schematic of minimal mechanisms of protein modification by a tight-binding affinity label, compared with a reactant that is not complementary to the protein target. The latter reaction is portrayed as a bimolecular reaction lacking both an intermediate and tight-binding interactions between the affinity group and protein.

Figure 8 shows schematically, the rudimentary properties of an affinity label, as well as a reactant that is not complementary to the protein target. The latter reaction is portrayed as a bimolecular reaction lacking both an intermediate and tight-binding interactions between the affinity group and protein.

For affinity labels, the minimal reaction mechanism is depicted as initial binding of the affinity label to the protein target, followed by a second step, in which an irreversible reaction ensues [13]. By virtue of complementarity between the affinity label's binding determinants and the target site, the affinity label pre-associates with the protein. Pre-association enables a facilitated reaction that exploits entropic and orientational effects. Such effects place the X-Y function in proper apposition for a reaction with a proximal nucleophilic group. Enzyme reactions that exploit such factors are thought to be accelerated by factors as high as 10^17^, compared to model chemical reactions [16, 17]. It might be expected that the contribution of these factors to reactions occurring at protein sites other than active-sites, may provide significant (albeit, more modest) rate increases over analogous reactions lacking such attributes [18].

From this perspective, the attributes of an affinity label embody, not only affinity and complementarity toward the target site, but also embrace an accelerated rate of labeling. Such rates are made manifest by comparing them to average labeling rates of other library members, and to a standard lacking a complementary group. Examination of affinity labels, in a well-defined series, indicates that as binding interactions become “tighter”, the labeling rates of a specific protein target increase [19–21]. If significant rate increments over average labeling rates signal site-selective reactions, a powerful basis exists for screening libraries for the discovery of affinity labels.

**Figure 9:**
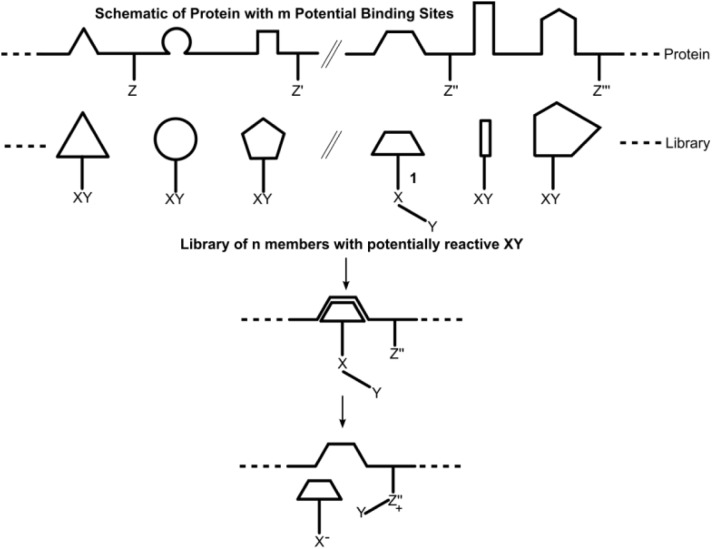
A protein of m sites, treated with a library of n molecular shapes representing potential affinity labels, is shown schematically. Library members seek access to any complementary sites on the protein, which may result in transfer of Y to a proximal nucleophile.

The strategy that we have employed in library design is depicted, schematically, in Figure 9. A protein of m sites is represented with each site possessing a defined structure, as indicated by the site's geometrical shape. A library of n molecules, schematically represented as diverse geometrical shapes attached to the same labeling entity, Y, is prepared. The protein is then contacted with the library and screened for incorporation of Y. The “winning” molecules are the fastest reacting molecules to label the protein.

It was our thought that significant rate variances for protein labeling might be observed within a collection of diverse, chiral, peptidyl moieties appended to the same reactive labeling entity. The notion of rate variances pertaining to competing chiral reactions has abundant precedents embedded in examples of competing reactions of diastereomers, which permeate the culture of organic chemical synthesis; chiral auxiliaries are prime examples of how chiral molecules can influence rates and bias reactions toward a particular stereochemical end [22].

We asked the question, whether any fast labeling pools could be identified that would ultimately reveal site-specific labelers by deconvolution, and provide the motivation for investigations in other library settings, where interpretation of kinetics would be more straightforward, e.g., spatially-separated libraries. In this preliminary study, using pooling, it is understood that myriad interactions are likely to occur, rendering interpretation of labeling rates extraordinarily challenging. Therefore, our goal was to ascertain whether significant positive deviations from average labeling rates could be observed, that might signal site-specific reactions. Although site-specific labeling may not be simply correlated with labeling rates, the notion that relatively fast rates (“kinetic affinity”) might be tied to site-specificity seemed reasonable, in analogy with high affinity/high specificity trends, which Eaton et al. have argued are correlated [10].

### Deconvolution can Track an Affinity Label in Kinetic Labeling Libraries

Treatment of HSA with each of the 81 pools produced variances in sort values as a measure of labeling rates that ranged from 0.6-3.5 after subtracting out the average value of 1.21 (Figure 3). It is noteworthy that the affinity labels herein differ from classical peptidyl affinity labels which link the peptidyl group to the protein product. By contrast, in our kinetic labeling libraries the affinity group is part of the leaving group and is extruded during the acylation step. Consequently, the variable peptidyl element could, in principle, be oriented distal from the protein, and not contact the protein in rate-determining steps. The observed rate variances, along with the disparate behavior between (7-E), and (9), containing an enantiomeric component, are in accord with the peptidyl group being an important determinant of rate and selectivity. Thus it is tempting to assume that such variances are intimately related to interactions between the peptidyl affinity group and the protein.

Since little was known about chemistries involving multicomponent systems, HSA was subjected to library pools for short contact times (1 min), to limit the possibility of secondary and multimeric reactions that might be anticipated in systems of this sort. Consequently, the addition of high concentrations of hydroxylamine, which react rapidly with thioesters, were employed to quench the acylation of HSA. It was expedient to also limit the size of the overall library (6561), so that the number of members (81) in each pool was kept sufficiently low, to minimize mixing, solubility, and aggregation problems, during the short contact times with protein [23].

Three parent pools (O_1_O_2_ = QE, RR, and FL) were noteworthy for their relatively high sort values. The FLXXE-NH_2_ pool was the clear outlier; members of this pool of 81, were resynthesized in a format involving nine pools of nine members, of general formula J-FLOXE-NH_2_ (5). Kinetic plots revealed a dichotomy between the pool denoted by J-FLYXE-NH_2_ (7) and the other eight (Figure 4). Indeed, all nine pools react more rapidly with HSA than the standard lacking an affinity group: but none of the pools substantially exceed the rate of labeling of the progenitor pool of 81 (J-FLXXE-NH_2_), except the sub-pool (7), defined by tyrosine at position 3, which may exhibit a burst as well.

Within the narrow range of rates, it appears that hydrophobic residues at position 3 (Y, F, L) produce the fastest labeling sub-pools of nine in this series. But it is clear that the rates of members of the J-FLYXE-NH_2_ (7) pool, contribute disproportionately to the relatively high labeling rate of the FLXXE-NH_2_ pool of 81.

The nine individual peptidyl thioesters constituting the J-FLYXE-NH_2_ pool (7) were synthesized and tested separately against HSA. The individual peptidyl thioesters reacted more rapidly with HSA, with relative rates varying between 3-13, relative to the standard (6). J-FLYEE-NH_2_ (7-E) reacts most rapidly with HSA and was the focal point of further studies under stoichiometric conditions.

### Site-specific labeling and the Locus of Attack

Human serum albumin contains 185 charged residues, and 59 lysines, which make it a formidable target, but one with numerous *binding* and *bonding* precedents. The literature to 1996 has been reviewed by Peters [15]. A major locus of attack has been Sudlow site I which is situated in the subdomain IIa of HSA [24, 25]. Three lysine residues, Lys-190, Lys, 195, and Lys-199, reside in Sudlow site I and have been implicated in various small molecule chemical reactions. The chemical behavior of Lys-199 has been attributed to an unusually low pK_a_ of 8 for Lys-199 [26].

Evidence for acylation (penicillins and β-lactams [27–30], aspirin [31–34]), Michael reaction (trans-4-hydroxy-2-nonenal [35]), and imine formation (pyridoxal phosphate [36], glucose [37, 38]) have been reported for lysines which reside in the Sudlow I site, with Lys-199 being a frequent point of attack. Additional products accompanied the adducts that were identified in these foregoing studies. Obvious conclusions that can be drawn are that the nature and distribution of the adducts formed are a function of time, concentration, and the medium in which the experiments are performed (e.g., buffer vs serum); a clear indication of the intrinsic chemistry between HSA and its co-reactant is best carried out in the absence of other ligands.

In the present study, we did not detect modifications of Lys 195, nor Lys-199. Not surprisingly, it seems clear from the various studies that albumin adducts are very much a function of specific structural features of the small molecule substrates [28, 29].

In the present, preliminary study of kinetic labeling libraries, it is gratifying that compelling evidence for site-specific labeling has been obtained with a modest size library of 6561 members, and a relatively short peptide of five amino acid residues. This study of irreversible labeling of a protein by a multicomponent system was unprecedented, so, a priori, it was not clear at the outset what the minimal length to achieve a successful hit might be and some arbitrary decisions governed the final design motifs. Since the library was screened using a split pool format after Furka, the number of mixture positions (x) and the number of distinct amino acids (Y) determine the number of members per pool (Y^x^). We chose, in this first study, to limit the number of amino acids employed in the construction of the library to nine, in order to restrict the number of members per pool to less than 100 members. Preliminary studies of pools beyond 100 members suggested that it would be more challenging to control mixing, solubility, initial kinetics, and to manipulate the screens. Given the short contact times between library and protein that need to be employed to limit labeling to a few per cent and avoid multiple labeling of the protein, we chose to limit the size of the pools to 81 members using only two mixture positions (XX).

For targeting established ligand binding sites, precedents from high throughput screening of synthetic combinatorial libraries show that a hexapeptide length [39, 40] is sufficient to achieve a number of “hits” for the discovery of tight-binding ligands. Intuitively, specific binding to sites other than the primary binding sites of proteins, followed by specific labeling of a fortuitously positioned amino acid residue, might be expected to be a much rarer event [41]. Therefore we chose to lengthen the basic peptidyl moiety by arbitrarily adding only a single defined amino acid at the C-terminal, so as not to overly bias the library, and bring it up to the pentapeptidyl level. The choice of glutamate was dictated by the fact that human serum albumin is well known to bind diverse molecules containing carboxylate moieties [15].

An amino acid of D-configuration was chosen to occupy one of the peptide positions for potential studies in vivo. Our expectation was that such peptides would be less susceptible to degradation than peptides containing only amino acids of L-configuration. D-amino acids were arbitrarily chosen to occupy an internal position, position 2.

In summary, the evidence clearly indicates that J-FLYEE-NH_2_ (7-E) has the attributes of an affinity label. Stoichiometric and site-specific biotin labeling by (7-E), taken together with its more rapid kinetics than other members of the pentapeptide library and the thioester standard, as well as the contrasting reactivity of (7-E) and (9), provide compelling support for this proposition. The data is totally consistent with the conclusion that HSA labeling by (7-E), within pools, is the result of a bimolecular reaction, and is not a consequence of a more complicated mechanistic scenario, unique to pooling.

This first study of kinetic labeling libraries, combining peptidyl functionality and biotin payloads, augurs well for the development of advanced methods of screening using solid phase techniques, mass spectrometry, and fluorescence detection, with more targeted and elaborate libraries (e.g., spatially-separated), possessing a range of functionality for innovative discovery of affinity labels.
